# Molecular Biomarkers of Canine Reproductive Functions

**DOI:** 10.3390/cimb46060367

**Published:** 2024-06-17

**Authors:** Marzena Mogielnicka-Brzozowska, Aleksandra Wiktoria Cichowska

**Affiliations:** Department of Animal Biochemistry and Biotechnology, University of Warmia and Mazury in Olsztyn, Oczapowskiego 5, 10-719 Olsztyn, Poland

**Keywords:** canine, semen, biomarkers, proteins, lipids, carbohydrates

## Abstract

The aim of the current study is to review potential molecular biomarker substances selected so far as useful for assessing the quality of dog semen. Proteins, lipids, carbohydrates, and ions can serve as molecular biomarkers of reproductive functions (BRFs) for evaluating male reproductive health and identifying potential risk factors for infertility or reproductive disorders. Evaluation of BRF levels in semen samples or reproductive tissues may provide insights into the underlying causes of infertility, such as impaired sperm function, abnormal sperm–egg interaction, or dysfunction of the male reproductive tract. Molecular biomarker proteins may be divided into two groups: proteins that are well-studied, such as A-kinase anchoring proteins (AKAPs), albumins (ALBs), alkaline phosphatase (ALPL), clusterin (CLU), canine prostate-specific esterase (CPSE), cysteine-rich secretory protein 2 (CRISP2), lactotransferrin (LTF), metalloproteinases (MMPs), and osteopontin (OPN) and proteins that are not well-studied. Non-protein markers include lipid-based substances (fatty acids, phosphatidylcholine), carbohydrates (glycosaminoglycans), and ions (zinc, calcium). Assessing the levels of BRFs in semen samples may provide valuable information for breeding management and reproductive assessments in dogs. This review systematizes current knowledge that could serve as a starting point for developing practical tests with the use of biomarkers of canine reproductive functions and their predictive value for assisted reproductive technique outcomes and semen preservation.

## 1. Introduction

Different reproductive biotechniques nowadays play a significant role in dog breeding. These biotechniques offer numerous benefits and advancements that enhance breeding programs and address various challenges faced by breeders. Fertility prediction and control in dogs using molecular biomarkers of reproductive functions (BRFs) is a key aspect of responsible breeding, health management, and population control. According to the Merriam-Webster Dictionary [1] definition, a biomarker is any biological component that can function as an indicator of a physiological pathway. Biomarkers are by definition objective, quantifiable rates of biological processes [2]. The development of simple molecular tests for advanced epididymal and ejaculated semen quality evaluation would be highly beneficial. Advanced molecular tests can more accurately assess the quality of semen samples (fresh and preserved), including sperm concentration, motility, morphology, and viability, leading to higher success rates in artificial insemination and natural breeding [3]. Additionally, knowing the exact quality of dog semen allows breeders to use it more effectively, ensuring that only high-quality doses are used for breeding, increasing the likelihood of conception. Additionally, modern semen evaluation can prevent costly unsuccessful breeding attempts and reduce the financial burden associated with poor-quality semen. Breeders can command higher prices for stud services if they can demonstrate that their sires produce high-quality ejaculates, backed by modern testing. Better semen quality evaluation improves the success rates of semen cryopreservation, ensuring that stored samples remain viable and effective for future use. High-quality semen is critical for successful in vitro fertilization (IVF) and subsequent embryo transfer, leading to better outcomes in reproductive technologies. With detailed information on quality of male used for reproduction, breeders can create more precise and effective breeding plans tailored to the specific needs and conditions of their breeding program. Modern semen evaluation with the use of molecular biomarkers contributes to a better understanding of canine reproduction and fertility, leading to further advancements in veterinary reproductive medicine. Implementing new technologies requires expanding knowledge and further research on canine semen storage techniques and procedures for handling semen. This is important for using valuable male reproductors, rare breeds of dogs and endangered canine species.

Thanks to access to increasingly modern research equipment, knowledge related to canine reproduction is developing in parallel to the expansion of areas of knowledge regarding human reproduction. The increasing demands of dog breeders and the changing living environment of these animals accompanying humans force a change in the approach of owners, veterinarians, and scientists to issues related to the reproduction of these animals. Modern technologies facilitate both detailed examination of the health of the reproductive organs and measurement of the quality of semen obtained from the male. Today, breeders expect accurate information on the health of their most valuable males because the success of their breeding and its profitability depends on it. Research has been ongoing for years to identify biomarkers of semen quality that could be easily linked to key parameters for the fertilization process and the specificity of the insemination dose. The most common substances contained in semen for this purpose include proteins, lipids, carbohydrates, and ions. All of these components are tightly interlinked, and their effective cooperation provides cellular signals required for sufficient sperm functional features. Relationships are sought between the level of a given substance in the animal’s reproductive organ fluids, sperm, blood, and selected semen quality parameters. Most often, the relationship between sperm motility, the condition of sperm plasma membranes, and DNA integrity is taken into account.

In canines, proteins from epididymal fluid or seminal plasma may coat sperm plasma membranes via phospholipid binding, exerting protective effects [4]. Additionally, these proteins, when bound, may also act as different ion carrier proteins or proteins with enzymatic functions [5,6]. Their role may be to alter ion concentrations in seminal fluid and spermatozoa, acting as decapacitation factors or protease inhibitors, as well as serine proteases affecting the activity of other proteins [6]. Moreover, these proteins might influence various aspects of sperm functions, for example, sperm membrane permeability and sperm motility, and they may modulate fertilization-associated events [7]. Lipids are involved in a wide range of reproductive events, from spermatogenesis and maturation to fertilization. As major compartments of sperm membranes, lipids maintain their integrity, control fluidity, and provide functional membrane microstructural domains and signaling molecules. Usually, different types of lipids do not function separately; rather, they act together in complex signaling pathways [8]. The maintenance of proper sperm function is also dependent on the balance between carbohydrates and ions, working intracellularly and extracellularly. Therefore, when searching for BRFs of dogs, it is necessary to take not only an individual approach but also a holistic, comprehensive approach to the analyzed molecules. Due to the growing interest in dog breeding over the years, as well as the dog being a model of the male reproductive system [9,10], it seems advisable to conduct a comprehensive examination of dog semen, including high-throughput OMICs (proteomics, lipidomics, metabolomics) and molecular biology tools, considering different variables such as age, breed, and pathological and environment conditions.

The aim of the current study is to review potential molecular biomarker substances selected so far as useful for assessing the quality of dog semen.

## 2. Protein Markers

Protein profiling of a reproductive tissue or a sperm cell, defined as proteomics, provides new possibilities and promising potential in dog reproduction research [11]. The detection and identification of protein biochemical properties, which influence their functional features and result in reproductive success, is important. Identification of proteins influencing sperm motility or sperm membrane integrity may be used in biomedical strategies for semen assessment, infertility treatment, and semen conservation improvement [12]. Some proteins that are abundant in semen or reproductive tissue were recognized many years ago in canines. Their functions in reproduction are well known. However, certain proteins have been newly discovered in canine semen and tissue and are not yet well-known; however, they possess a potential that needs to be recognized more widely. In this article, the characteristics of both types of proteins are provided, and their potential as markers in dog breeding is explored.

### 2.1. Well-Studied/Highly Abundant Proteins

Some proteins have been well studied and are recognized as being correlated with canine semen quality parameters.

A-Kinase Anchoring Proteins (AKAPs) share the property of binding to the regulatory subunit of protein kinase A (PKA) [13]. ProAKAP4 (Pro A-Kinase Anchoring Protein 4) is expressed as a precursor of the AKAP4 (A-Kinase An-choring Protein 4) [14]. ProAKAP4 and AKAP4 are involved in a signaling cascade including adenosine monophosphate cyclic, protein kinase A, tyrosine kinase, and phosphatase [15]. These proteins are actively phosphorylated on their tyrosine and serin residues during capacitation, which allows us to consider them as biomarkers of the capacitation status [16,17]. ProAKAP4 is produced during the round spermatid stage and is incorporated into the fibrous sheath of the developing sperm flagellum [18]. ProAKAP4 is cleaved during the condensed spermatid stage, resulting in its conversion into active AKAP4 [13,18]. It has been described as a functional marker of sperm in mammals [19,20]. AKAP4 is associated with good-quality spermatogenesis and is involved in the regulation of sperm motility [14,21]. Le Couazer and Bencharif (2021) [14] showed, for the first time, the localization of proAKAP4 and AKAP4 in the fibrous sheath of the dog’s spermatozoid and their expression in the spermatic part of the dog’s ejaculate (not in the urethral or prostatic fractions) [22]. Additionally, in humans, the absence or weak expression of proAKAP4 and AKAP4 was associated with poor sperm motility [23]. There are kits (Dog 4MID^®^ kits, 4BioDx, Lille, France) available on the market allowing for objective validation of semen quality by quantifying the proAKAP4 in dog semen. The translation of fundamental discoveries around proAKAP4 and AKAP4 semen quality markers into a practical tool (such as 4MID^®^ kits) is of great importance in breeding and veterinary practice [20]. However, further detailed research is needed on the expression of proAKAP4 and AKAP4 in canine semen and its association with canine reproductive functions.

Albumins (ALBs) are proteins with a molecular mass of about 65 kDa [24]. In addition to being the main component of blood serum, they are also known to be an important component of the seminal plasma of the canine [25] epididymal spermatozoa [26] and epididymal fluid [27]. A semi-quantitative analysis of ALBs in canine epididymal fluid showed its higher abundance in the good sperm motility group [27]. In the reproductive system, they are mainly secreted by the testes, epididymides, and prostate [24]. The addition of heterogenous bovine serum albumin (BSA) to the ejaculated canine spermatozoa preserves sperm viability and function [28] and improves sperm capacitation and acrosome reaction [29]. Studies of homologous ALBs (found in the seminal plasma) have shown that they are implicated in fertilization-associated events, such as sperm capacitation [30] and zona pellucida penetration [31]. The seminal plasma ALB content is highly correlated with the motility parameters of animal spermatozoa [32]. The exact mechanism of action of ALBs is not known. It has been postulated that they may act via two mechanisms. One is via their antioxidant properties and the other is by binding bivalent cations to regulate their number in the reproductive fluids [5,33]. Even a simple mechanism of binding seminal plasma or epididymal fluid ALBs to the plasma membrane of ejaculated or epididymal spermatozoa plays a protective role in sperm motility [27,32,34]. Due to their antioxidant properties, ALBs can absorb lipid peroxides from reproductive fluids, which contribute to their protective effect on the structures involved in sperm motility and sperm membrane integrity [25]. It has also been demonstrated that ALBs possess a strong ability to bind zinc ions, thus reducing their concentrations in the spermatozoa and enhancing the motility of the cells [32].

Alkaline phosphatase (ALPL) is a dephosphorylation enzyme present at very high concentrations in canine reproductive tracts [35]. In dogs, most of the ALPL is produced in the epididymis, but it is also found in seminal plasma [36,37]. It also plays a role in the transport of sugars and other organic molecules across biological membranes [38]. Its activity has been demonstrated in cytoplasmic droplets, which raises the hypothesis that ALPL catalyzes dephosphorylation and transport of phosphate groups between the epididymal sperm and epididymal fluid. ALPL might be a potential biomarker for prostate and testicular cancer [39]. This enzyme inhibitor can reduce the growth and invasion of cancer cells [39]. Measurement of the enzymatic activity of ALPL in the canine seminal plasma has been used for the diagnosis of incomplete ejaculation or azoospermia in dogs [38], and a reduced concentration of ALPL in the seminal plasma suggests bilateral obstruction of the vas deferens or epididymis [40].

Clusterin (CLU) is a common SP protein in many species [41,42,43]. CLU is an extracellular chaperone secreted in high amounts by epididymides and testes [44,45]. Its presence in the canine epididymal sperm and fluid has been established [26,27]. CLU might be adsorbed from the epididymal fluid and cover epididymal sperm plasma membranes, positively influencing sperm motility [27]. However, it may also be an indicator of low semen quality because secretion of this protein is enhanced in case of cellular damage or heat shock [42,46,47]. It is overexpressed in several human cancers, such as prostate cancer [48]. Morphologically defective sperm extensively bind CLU to its plasma membrane [49]. This protein has a role in inhibiting cell apoptosis, mediation of female tolerance to seminal antigens, and sperm maturation processes [50]. It also participates in membrane remodeling and DNA reparation [48]. CLU participates in sperm maturation by affecting lipid transport and membrane remodeling [48].

Canine prostate-specific esterase (CPSE) accounts for more than 90% of the proteins secreted by the prostate and about 30% of the canine SP proteins. The enzyme has a molecular weight of 29 kDa and it can be dissociated into two subunits with molecular masses of 12–14 kDa and 15 kDa [51]. The CPSE identified in canine seminal plasma is a multifunctional protein due to its zinc-binding and phosphorylcholine-binding properties [4,6]. The enzyme has been detected in the post-acrosomal region and sperm tail of ejaculated spermatozoa of dogs [51]. It can bind to phosphorylcholine of the sperm plasma membrane and coat ejaculated spermatozoa, which could be implicated in sperm fertilization-related events. It is noteworthy that CPSE is utilized as a marker of glandular secretion and serves as a promising diagnostic tool for non-neoplastic canine prostatic disorders [40]. Interestingly, CPSE is an enzyme homologous to human prostate-specific antigen (PSA), which is a direct indicator of the development of prostate cancer. Both CPSE and PSA are members of the kallikrein family, a group of serine proteases [52]. This family also includes the horse prostate kallikrein (HPK), which, like human PSA, has the ability to bind zinc and mercury ions [52]. Findings by our team confirmed that CPSE can bind zinc ions, a property previously demonstrated only by Isaacs and Coffey (1984) [51]. This underscores the role of zinc ions in the proteases’ activity in dog semen.

Members of the cysteine-rich secretory protein (CRISP) family have been found in spermatids [53,54] as well as in the acrosome and tail of ejaculated sperm [55,56,57,58]. CRISP expression is high in the testis, and it is present at the junction between germ and Sertoli cells within the seminiferous epithelium [55,56,57,58]. CRISP2 specifically regulates calcium flow through ryanodine receptors [59,60], is implicated in cell–cell adhesion, and is capable of binding to steroids [61,62]. A decrease in CRISP2 content in sperm is associated with infertility in humans [53,54] and horses [63]. It was found that the content of this protein in the canine epididymal spermatozoa was low in very young and senile dogs [26].

Lactotransferrin (LTF) is an iron-binding protein that regulates the availability and catalytic activity of iron [64]. In canines, LTF was found in seminal plasma, and further studies showed that it originates in the epididymis [4,27], similar to other animal species e.g., mice, boars, and stallions [65]. It was also found to be a component of canine epididymal sperm [26,34]. LTF binds to phosphorylcholine-containing phospholipids of the sperm plasma membrane in dogs [4]. LTF has antibiotic properties in the reproductive system, conveyed by its ability to sequester iron and prevent the harmful effects of pathogens on spermatozoa [64,65]. Additionally, LTF can bind lipopolysaccharides, heparin, glycosaminoglycans, DNA, and ions like Mn^3+^, Co^3+^, Cu^2+^, and Zn^2+^ [65]. The exact function of LTF in the canine reproductive system is not known, but since it is present in high amounts, it must be important for canine reproductive functions. The addition of LTF to a cryoprotective extender can significantly improve the function of frozen ram sperm [66].

Metalloproteinases (MMPs) are mostly expressed in the epididymis, where they contribute to the modification of the sperm membrane and the regulation of sperm maturation and storage [67]. MMPs aid in the processing of proteins on the sperm surface, which is crucial for sperm function and fertilization [68]. MMP-2, MMP-9, proMMP-2, and proMMP-9 were identified in canine seminal plasma [69]. Saengsoi et al., (2011) [69] suggested that higher activation of proMMP-2, proMMP-9, and MMP-9 may be caused by an abnormal spermatogenesis process, whereas MMP-2 may benefit sperm motility and viability. Studies have shown associations between MMP amounts in seminal plasma and parameters such as sperm concentration, motility, and morphology [70]. Zinc-binding abilities have also been demonstrated for MMPs [70]. The levels of MMPs and their tissue inhibitors are correlated with sperm motility and sperm DNA fragmentation in men [71,72]. Alterations in MMP expression or activity may indicate abnormalities in sperm function [71,72]. MMPs have been proposed as predictive biomarkers for the success of assisted reproductive techniques (ART), such as IVF and intracytoplasmic sperm injection (ICSI) [73]. Assessing MMP amounts in semen or reproductive tissues may help predict fertilization outcomes and the likelihood of successful embryo implantation.

Osteopontin (OPN) is expressed in the male reproductive tract, including the testes and epididymis [74]. It has been implicated in various aspects of sperm function, such as sperm maturation, motility, and capacitation, which are essential for successful fertilization. Lower OPN concentrations have been linked to poorer sperm motility and morphology [75]. OPN levels in seminal plasma vary depending on factors such as breed, age, and reproductive health status [76]. OPN has been identified in the seminal plasma of dogs, indicating its secretion by the male reproductive tract and its presence in the ejaculate [75,76,77]. Erikson et al., (2007) [78] suggested that OPN localized to the post-acrosomal region on sperm membranes may participate in bovine fertilization by interacting with egg integrins. OPN may also regulate sperm adhesion to the female reproductive tract, their migration towards the egg, and sperm–oocyte interaction [79,80]. OPN has been shown to play a major role in tumorigenesis, tumor invasion, and metastasis, with reported associations with breast, prostate, and ovarian cancer [81].

The prostaglandin-H2 D-isomerase, also known as prostaglandin-D2-synthase (PTGDS) or lipocalin-type prostaglandin-D-synthase, is an enzyme that converts the cyclooxygenase product of prostaglandin H2 (PGH2) to prostaglandin D2 PGD2 [82]. It is an enzyme that binds small non-substrate lipophilic molecules such as retinoids [83]. It also acts as a scavenger of harmful hydrophobic molecules [83]. Moreover, lipocalins bind to specific cell-surface receptors and may form macromolecular complexes [84]. PTGDS also affects plasma membrane permeability, resulting in changes in the input of ions from the outside, which may be correlated to the regulation of sperm motility and access to Ca^2+^ ions by the sperm cell [83,85,86]. PTGDS has been found in the epididymal fluid and seminal plasma of rams [86] and cats [87]. A positive correlation between PTGDS content in human sperm and cell progressive motility has been shown [88]. PTGDS is interconnected with several essential proteins involved in sperm metabolism, such as CRISP2 and lipocalin cytosolic FA-bd domain-containing protein (LCNL1). However, the exact function of PTGDS in sperm metabolism is unknown. A proteomic study revealed that PTGDS is present in greater abundance in dog epididymal fluid surrounding sperm showing good motility [27].

**Figure 1:**
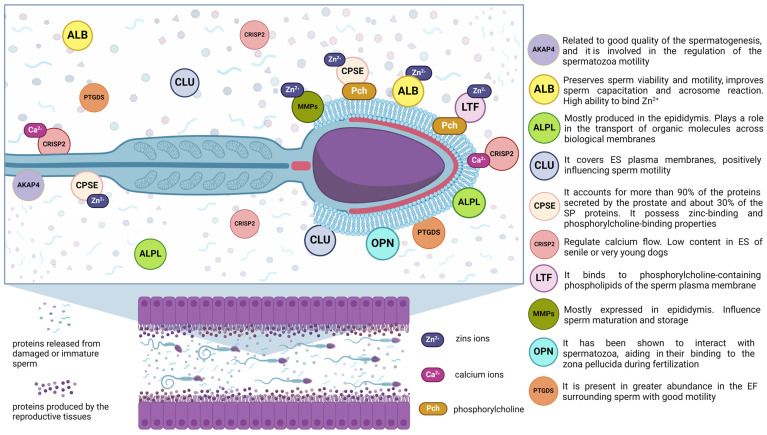
Well-studied/highly abundant molecular biomarker proteins that coat canine sperm and change its surface properties, biochemistry, and metabolism. AKAP4—A-kinase anchoring protein; ALB—albumin; ALPL—alkaline phosphatase; CLU—clusterin; CPSE—canine prostate-specific esterase; CRISP2—cysteine-rich secretory protein 2; LTF—lactotransferrin; MMPs—metalloproteinases; OPN—osteopontin; PTGDS—prostaglandin-H2 D-isomerase; SP—seminal plasma; EF—epididymal fluid; ES—epididymal spermatozoa. Created with BioRender.com.

Well-studied molecular biomarker proteins that coat canine sperm and change its surface properties, biochemistry, and metabolism are shown in Figure 1.

### 2.2. Poorly Studied/Newly Recognized/Low-Abundance Proteins

Canine reproductive tissues, fluids, and sperm contain newly recognized proteins that could serve as potential protein markers for semen quality.

Acrosin binding protein (ACBP) is a calcium-binding protein detected in the semen of dogs (Bernese Mountain) [29]. The ACBP is a binding protein for both the precursor and intermediate forms of serine protease acrosin, a protein that is specifically localized in the acrosomes of germ and sperm cells [89]. ACBP improves sperm capacitation, acrosome reaction, and semen quality [29]. ACBP can be used as a molecular marker for pachytene spermatocytes, and for round, elongating, and elongated spermatids. ACBP can be used to monitor either normal spermatogenesis in the testicular tissues, or germ cell development in vitro [90]. ACBP may be a good marker for predicting boar sperm freezing capacity [91].

Actin binding protein (ACTB) is a structural protein that builds the cytoskeleton and is present in the flagellar and acrosomal membranes of spermatozoa. It is also responsible for cell volume changes [92,93]. Based on its location, its role was proposed to be in sperm capacitation and motility [92,93,94]. ACTB was previously found in the canine epididymal spermatozoa, and its expression was correlated with the dog’s age [26]. Additionally, it was found in canine epididymal fluid, and it is highly abundant in semen with good sperm motility [27]. The exact mechanism of ACTB’s influence on sperm motility needs to be evaluated in the future.

Abnormal spindle-like microcephaly-associated protein homolog (ASPM) is expressed in a variety of embryonic and adult tissues and is upregulated in cancer [95]. It has been found in canine epididymal spermatozoa [26]. ASPM misfunction affects chromosome segregation, which leads to reduced ability of fetal stem cells to produce neurons [95]. ASPM plays a role in sperm flagellar function [96].

Caspase recruitment domain containing protein 6 (CARD6) is found in the canine epididymal fluid [27]. Its function is to regulate cell proliferation, immune response, and cell apoptosis [97]. CARD6 may be involved in microtubular transport mechanisms, and proteins that interact with it may be targeted to the microtubule-organizing center, resulting in either their inactivation or translocation [98].

Hyaluronoglucosaminidase (CEMIP), also named KIAA1199, is a unique protein found in the epididymal spermatozoa of young dogs [26]. It is expressed in the human testis, and its biological role in cancer biology has been studied in humans [99]. It has not been described in other species, including dogs. This protein causes significant changes in cell morphology and actin cytoskeletal dynamics. It exerts its effects through regulation of the canonical Wnt and P38/MAPK signaling pathways [100] and the expression of CEMIP may be regulated depending on cell mortality rather than cell age [99].

Epididymal sperm-binding protein 1 (ELSPBP1) was first described in humans and dogs as a sperm-binding protein of epididymal origin that binds to the spermatozoa during their transit through the epididymis [101,102]. Since then, orthologs have been identified in horses [103], pigs [104,105], and bovines [102]. More recently, ELSPBP1 was shown to negatively correlate with bull fertility [106] and was associated with the dead sperm population [107]. It has been found in epididymal spermatozoa, and its presence is age-dependent [26].

The function of a family with sequence similarity 135 member A (FAM 135A) is the regulation of cellular proliferation, cell differentiation, development, and growth control [108]. Expression of the protein was observed in the testis, epididymis, prostate, and seminal vesicles in humans [109]. This protein has also been found to be a unique protein in the epididymal spermatozoa of older dogs [26].

N-acetylgalactosaminyltransferase-like proteins (GALNTL) were found in male reproductive organs in rodents and cattle [110,111,112,113]. In canines, it has been shown that polypeptide N-acetylgalactosaminyltransferase 6 (GALNT6) is important for epididymal sperm motility [27] and participates in mucin-type-O-glycan biosynthesis [26]. We can point out more reports about GALNTL5. In mice, it is localized in the head–tail coupling apparatus of cauda epididymal spermatozoa [112], and its expression is positively correlated with sperm motility [114].

Lipocalin cytosolic FA-bd domain-containing protein (LCNL1) is a member of the lipocalin family. These proteins transport or store small molecules, such as vitamins, hormones, and secondary metabolites [115]. LCN family proteins are important for sperm maturation, and they are expressed in different regions of the epididymis [116,117]. LCNL1, present in canine epididymal fluid, is important for sperm motility, although the exact mechanism is unknown [27].

Cystatin domain-containing protein (LOC607874) may serve as potential biomarker of canine aging [26]. A record for *Canis lupus familiaris* LOC607874 mRNA is found in the National Center for Biotechnology Information (NCBI). The cystatin-related epididymal-specific (*CRES*) gene is almost entirely restricted to the epididymis and much less is expressed in the testis, mainly in spermatids [118]. A *CRES* gene, which shares similarities with well-known protein inhibitors called cystatins, was found in the mouse epididymis [119]. This protein is unique to the epididymal spermatozoa of older dogs [26] and its presence in the epididymal fluid has been associated with poor sperm motility [27].

Niemann-Pick type C2 protein (NPC2), intracellular cholesterol transporter 2, also called epididymal secretory protein E1, is found in the canine epididymal spermatozoa [26]. In canines, epididymal secretory protein E1 (CE1/NPC2) is encoded by genes similar to those in humans [120]. The CE1 protein is a highly abundant, conserved, secretory protein [121] and its mRNA is found in large amounts in the epididymal duct epithelium, while the protein is found in the duct lumen [122]. Recently, *CE1* has been identified as a gene that is important in the etiology of Niemann-Pick type C disease [123]. This protein is involved in cholesterol efflux from lysosomes [123]. In human ejaculated spermatozoa, a human sperm antigen (HE2), human homolog of CE1, was found in the acrosome and equatorial region of the cells [124]. NPC2 epididymal sperm-membrane-coating ability has been suggested [34] because similar results were shown by Araujo et al. [29,125], who found NPC2 to be an ejaculated sperm component.

Olfactomedin 4 (OLFM4) is an olfactomedin domain-containing glycoprotein [126]. OLFM-1, -2, -3, -4 are known to regulate cellular growth, differentiation, and pathological processes [127]. The absence of *OLFM4* gene expression is associated with the progression of human prostate cancer [128]. OLFM4 is found in human spermatozoa [129]. The OLFM-4 precursor is found in human epididymosomes [130]. The role of OLFM4 in sperm physiology is unknown. This protein was found to be expressed in the epididymal fluid of dogs and was correlated with good sperm motility [27].

Pleckstrin homolog, MyTH4, and FERM domain-containing H1 (PLEKHH1) is a protein constituent of the cytoskeleton with a role in intracellular signaling [131]. It binds phosphatidylinositol lipids within biological membranes and plays a role in recruiting proteins to membranes. PLEKHH1 expression is found in human testis, epididymis, seminal vesicles, and prostate [132]. This protein has also been found in the canine epididymal spermatozoa [26]; however, its function has not yet been established.

The tubulin (TUBB) family proteins are associated with structural cell organization and flagella movement [29,133,134,135]. These proteins have also been identified in the sperm membrane of Morada Nova rams [136] and mice [137]. In humans, tubulins are associated with low sperm motility [138,139]. A study by Araujo et al., (2022) [29] indicated a high abundance of tubulin alpha-3E chain in Maremmano-Abruzzese Sheepdogs and tubulin alpha-3 chain in Bernese Mountain Dogs [29]. Tubulins in seminal plasma are linked to damaged sperm in the ejaculate, possibly due to sample handling [140].

**Figure 2:**
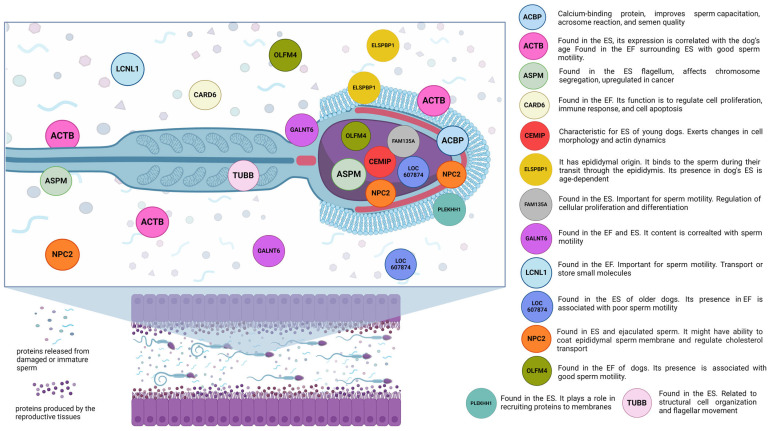
Poorly studied/newly recognized/low-abundance molecular biomarker proteins in canines that change sperm biochemistry and metabolism. ACBP—Acrosin binding protein; ACTB—Actin binding protein; ASPM—Abnormal spindle-like microcephaly-associated protein homolog; CARD6—Caspase recruitment domain containing protein 6; CEMIP—Hyaluronoglucosaminidase; ELSPBP1—Epididymal sperm-binding protein 1; FAM135—A family with sequence similarity 135 member A; GALNT6—Polypeptide N-acetylgalactosaminyltransferase 6; LCNL1—Lipocalin cytosolic FA-bd domain-containing protein; LOC607874—Cystatin domain-containing protein; NPC2—Niemann-Pick type C2 protein; OLFM4—Olfactomedin 4; PLEKHH1—Pleckstrin homolog, MyTH4, and FERM domain-containing H1; TUBB—Tubulin; EF—epididymal fluid; ES—epididymal spermatozoa. Created with BioRender.com.

Poorly studied/newly recognized/low-abundance molecular biomarker proteins in canines, which represent less studied proteins that change the biochemistry and metabolism of sperm, are shown in Figure 2.

### 2.3. Antioxidant Enzymes

Glutathione peroxidase (GPX) is a well-known, highly abundant protein in the canine epididymis [141]. GPX5 is highly expressed in the epididymis and is then secreted into the lumen. Its role is to protect sperm from lipid peroxidation [141]. GPX is found in, among others, bull, boar, and dog seminal plasma [142,143,144] and in boar epididymal spermatozoa [144]. This protein also participates in important metabolic pathways in feline seminal plasma [87]. The above-mentioned protein works in a SOD/CAT system. Antioxidant enzyme activities of mammalian spermatozoa comprise those provided by superoxide dismutase (SOD) and GPX. SOD has been shown to play a key role in the intracellular scavenging of superoxide anions [145]. The superoxide anion generated by the inner mitochondrial membrane is dis-mutated to hydrogen peroxide by the Mn-SOD present in the mitochondrial matrix and by the Cu/Zn-SOD present in the cytosol. In turn, hydrogen peroxide is eliminated by either GPX or catalase to avoid cell damage. Because it has been shown that sperm from most mammalian species lack catalase activity, sperm mostly rely on GPX activity to eliminate intracellularly generated hydrogen peroxide [146]. It has been reported that both SOD and GPX play a central role in protecting mammalian sperm against oxygen-radical-induced damage leading to motility loss [146]. SOD could be a valid marker for resistance to cryodamage because the time observed for complete loss of motility due to lipoperoxidative damage to sperm under experimental conditions is strongly correlated with the SOD activity of the sperm sample [147]. A study of dogs confirmed the presence of endogenous antioxidants in the seminal plasma of pre-spermatic, spermatic, and post-spermatic fractions, with SOD representing the major enzymatic antioxidant in all dog ejaculate fractions, whereas GPX activity was present in the sperm-rich and post-spermatic fractions, while CAT activity was deficient [148]. Another study [149], however, demonstrated CAT activity in dog ejaculates and showed that the addition of SOD and CAT in the dilution extender of canine semen improved sperm quality. The addition of a GPX and SOD combination to dog semen can protect sperm viability and DNA integrity [150]. The addition of SOD, CAT, and GPX in the extender allows the preservation of semen quality in cold storage for both fertile and hypofertile dogs [151].

**Figure 3:**
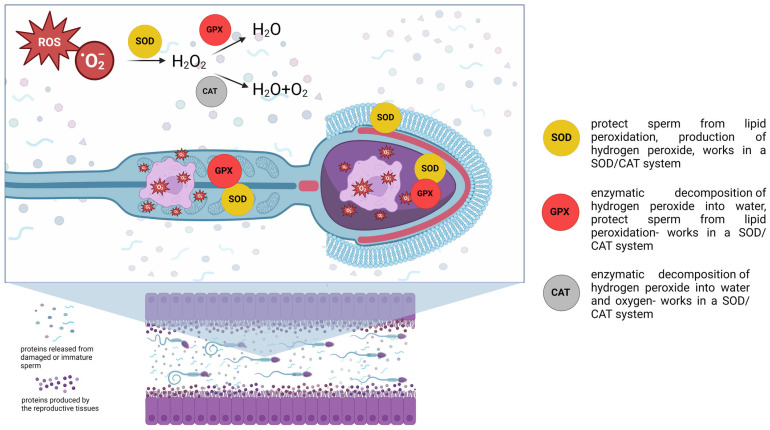
Antioxidant enzymes that function as molecular biomarkers and participate in sperm biochemistry and metabolism in canines. SOD—superoxide dismutase; GPX—glutathione peroxidase, CAT—catalase, ROS—reactive oxygen species.

Antioxidant enzymes functioning as molecular biomarkers that participate in sperm biochemistry and metabolism in canines are shown in Figure 3.

Table 1 contains a summary of the most promising protein molecular biomarkers of canine reproductive functions.

## 3. Non-Protein Markers

### 3.1. Lipids

Lipids are essential building compartments of cells, including most of all their plasma membranes, the endoplasmic reticulum, the nuclear membranes, endosomes, and lysosomes [153]. Lipids form highly specialized sperm plasma membranes. Phospholipids, fatty acids, glycolipids, and neutral lipids (such as cholesterol and diacylglycerols) are among the lipids found in plasma membranes [8,154]. Changes in lipid composition within membranes generally correspond to their physiological needs [8]. The sperm plasma membrane’s lipid profile varies during sperm transfer along the epididymal tract [155]. This facilitates the preservation of cellular stability and integrity, which are crucial for preventing sperm damage, as well as the progressive acquisition of functional competence by sperm, such as motility and the ability to fertilize [156]. Lipid reorganization of the sperm plasma membrane during sperm maturation occurs due to the region-specific environment surrounding sperm. Therefore, remodeling of the sperm plasma membrane’s lipid profile is influenced by substances present in the epididymal fluid [157,158,159]. In relation to the above findings, the arrangement of fatty acids in the sperm membrane appears to be a significant factor that could potentially elucidate the quality of sperm, including their cryotolerance [160,161,162]. Only a few studies have been conducted on canine semen’s lipid composition and its impact on semen quality.

#### 3.1.1. Fatty Acids

Angrimani et al., (2017) [159] identified differences between fatty acid profiles of epididymal spermatozoa and epididymal fluid derived from different regions of canine epididymis (caput, corpus, cauda). The authors study also showed that the fatty acids content correlate with sperm motility parameters and sperm plasma membrane integrity. Díaz et al., (2014) [163] identified canine seminal plasma fatty acids. Lucio et al., (2017) [164] conducted an analysis of the lipid profile of dog-ejaculated sperm, which the authors used to identify lipid markers of sperm motility.

Results obtained by Angrimani et al., (2017) [159] indicate a progressive increase in the dog sperm motility and plasmatic membrane integrity from caput to cauda epididymis. The same pattern was also observed for the concentrations of fatty acids (saturated, monounsaturated, and polyunsaturated).

##### Saturated Fatty Acids

According to Angrimani et al., (2017) [159], epididymal sperm had a higher concentration of saturated fatty acids (SFAs) compared to the epididymal fluid. Among fatty acids in canine seminal plasma, SFAs were observed to be the most abundant [163]. Caprylic and stearic fatty acids are the most abundant SFAs found in cauda epididymal sperm, as identified in a study by Angrimani et al., (2017) [159], while palmitic and stearic fatty acids are the most abundant in canine seminal plasma, according to Díaz et al., (2014) [163]. Caprylic fatty acid plays a dual role in the sperm membrane by exhibiting both bactericidal properties and protective functions, as per a study by [165]. Additionally, caprylic fatty acid was found to be effective in maintaining the viability of rooster sperm during in vitro storage when used as an extender [166]. It has been shown that stearic fatty acid regulates sperm function and provides the energy necessary for sperm motility and metabolism [167]. However, stearic fatty acid was also identified in human spermatozoa and seminal plasma and was negatively correlated with sperm motility [168]. High levels of stearic fatty acid were observed in lipid profiles of asthenozoospermic spermatozoa [169,170,171]. In humans, a positive correlation was observed between palmitic fatty acid and both sperm concentration and total sperm count, suggesting its potential significance in sperm production [172].

##### Monounsaturated Fatty Acids

For monounsaturated fatty acids (MUFAs), pentadecenoic fatty acid concentration was higher in the dog cauda epididymal sperm and fluid compared to epididymal caput and corpus, and the concentration increased from caput to cauda epididymal regions [159]. Pentadecenoic fatty acid has also been identified in canine seminal plasma, albeit in small amounts [163]; however, knowledge about its influence on epididymal sperm maturation and further semen quality is lacking. Oleic fatty acid is a characteristic component of the seminal plasma in both canines [163] and humans [168,173]. This fatty acid, along with stearic fatty acid, is associated with sperm metabolism and has a positive effect on both the motility and viability of boar spermatozoa [174].

##### Polyunsaturated Fatty Acids

Among polyunsaturated fatty acids (PUFAs), docosahexaenoic acid (DHA) is present in the dog cauda epididymal spermatozoa [159]. Cauda epididymal fluid also contains higher DHA levels than the remaining regions of the epididymis, although it was lower than in the dog cauda epididymal sperm [159]. The contents of DHA in mammalian sperm differ among species and range from low percentages in rats and rabbits (1%) to almost half of the total content of PUFAs in human sperm [160,175,176,177]. Waterhouse et al., (2006) [178] suggested that, when freeze-thawed, boar sperm with higher levels of DHA in their membranes were more cryotolerant, which was attributed to the higher membrane fluidity. Docosahexaenoic acid adds unique fluidity to the sperm plasma membrane [179], thus affecting its integrity, which is essential for sperm motility and acrosome reaction [179,180]. Moreover, DHA positively influences testicular testosterone secretion and enhances sperm antioxidant capacity [181]. A positive correlation between DHA in the sperm plasma membrane and sperm motility in boars was demonstrated [182], and a low content of this fatty acid was observed in human asthenospermic semen [160]. Based on the above findings, Angrimani et al., (2017) [159] proposed that increasing DHA concentration in the epididymal sperm and fluid is important for the final steps of epididymal maturation in dogs due to its direct involvement in fertilization.

It should be noted that the highest concentration of individual fatty acids occurred both in the sperm and fluids derived from the cauda epididymis—the place where sperm achieve the highest maturity level. Changes in the structure of the sperm plasma membrane’s lipids occur during sperm transfer through the epididymis. These changes are associated with the binding of fatty acids as well as other components, such as proteins, from the epididymal fluid [44,155]. This suggests that during epididymal maturation, the sperm takes up essential lipids, which may be vital for acquiring functional features required for its mission in the female reproductive tract. The highest concentration of fatty acids in the dog cauda epididymal sperm may contribute to the enhanced stability of the cell membrane [159]. Sperm cells are highly susceptible to oxidative stress due to the concentration of PUFAs found within the plasma membranes [183]. Unsaturated fatty acids add elasticity and fluidity to the plasma membrane, which are crucial for the proper development of sperm functional features such as motility [184]. However, their presence stimulates free radical activity and, thus, lipid peroxidation, which are the main causes of membrane disintegration [185]. A higher concentration of SFAs may reduce sperm damage during storage at low temperatures [176].

#### 3.1.2. Phospholipids

Among the group of phospholipids, very early reports have shown that the dog spermatozoa contain large amounts of phosphatidylcholine, phosphatidylethanolamine, sphingomyelin, ethanolamine plasmalogen, and choline plasmalogen [186]. Phosphatidylcholine and phosphatidylethanolamine were identified in the motile spermatozoa and less frequently in the spermatozoa of asthenozoospermic individuals [164]. The reduction in phosphatidylcholine and phosphatidylethanolamine content in the sperm of asthenozoospermic individuals stems from the extensive degradation and impairment of PUFAs caused by oxidation induced by free radicals [170,187]. Canine sperm motility is indicated by the lipid markers phosphatidylcholine and phosphatidylethanolamine, according to the above-mentioned reports. Interestingly, phosphatidylethanolamine in the sperm membranes has the capacity to bind to lipocalin 2 in the female reproductive tract. In turn, lipocalin 2 can stimulate the rearrangement of lipid rafts and the efflux of cholesterol [188].

##### Plasmalogens

Among the group of lipids, plasmalogens are of great interest based on their protective functions against oxidative damage in sperm. Shan et al., (2021) [8], in their thorough review, explained the mechanism of plasmalogen antioxidant properties. Moreover, in canines, plasmenyl phosphatidylcholine (40:5) and plasmanyl phosphatidylcholine (40:4) were reported as lipid sperm motility markers [164] due to their increased content in motile sperm and cells with the lowest percentage of acrosomal membrane damage. These findings might provide evidence that the presence of plasmalogens in the motile sperm may be part of a protective mechanism protecting the sperm plasma membrane against lipid peroxidation and preventing damage induced by reactive oxygen species (ROS) [164].

##### Sphingomyelins

Sphingomyelins are part of biological membranes [189], including the sperm plasma membrane [190,191], and function as bioactive lipids [189]. Sphingomyelin has an affinity for cholesterol [192]. Interestingly, this combination may generate low-density lipid rafts in the plasma membrane, thereby modifying the lateral arrangement of proteins [193]. An early study by Darin-Bennet et al., (1974) [186] showed that dog spermatozoa contain large amounts of sphingomyelin; however, the latest reports on this matter do not indicate the significance of sphingomyelin in dog’s reproductive functions.

##### Phosphorylcholine

The coating on the sperm plasma membrane is formed by phosphorylcholine moieties of phospholipids [194]. Protein complexes with the involvement of phosphorylcholine moieties also form the coating of the dog sperm plasma membrane during ejaculation and protect the spermatozoa against the adverse conditions in the female reproductive tract, as well as against cold shock during semen freezing [4,195]. These findings suggested that the attachment of phospholipids containing phosphorylcholine is an important mechanism of protein coating on the dog sperm plasma membrane [4,195]. Mogielnicka-Brzozowska et al., (2017) [4] have shown that the phosphorylcholine-binding proteins (PchBPs) in the canine seminal plasma are high molecular weight aggregates. Mass spectrometric analysis of canine seminal plasma PchBPs identified five different types of proteins: LTF, ALB, PTGDS, CPSE, and lipocalin-like 1 protein isoform.

#### 3.1.3. Cholesterol

Cholesterol is an important structural component maintaining the stability and fluidity of the cell membrane [196]. Cellular efflux of cholesterol is required to maintain homeostasis and is an essential step during the capacitation of spermatozoa [197]. Cholesterol is the precursor of steroid hormones, such as testosterone, produced by the Leydig cells, and plays an important role in normal spermatogenesis [198]. Cholesterol concentration in spermatozoa was shown to be associated with cryotolerance, motility, and morphology of sperm cells. Therefore, cholesterol in sperm cells provides protection against sperm damage induced by freeze/thaw processes [199]. Canine sperm membranes are not highly sensitive to cold damage due to their relatively high cholesterol:phospholipid ratio and the PUFA content of the membrane [200]. Schäfer-Somi and Palme (2016) [25] showed that in dogs, higher concentrations of cholesterol are found in the seminal plasma of ejaculates with good freezability than in the seminal plasma of ejaculates with bad freezability. The removal of seminal plasma from good-quality ejaculates decreased post-thaw motility, increased the percentage of morphologically abnormal sperm, and increased post-thaw DNA damage [201]. The movement of cholesterol from the sperm plasma membrane involves specific molecules with a high affinity for cholesterol, for example, NPC2, which is closely associated with the lipid structure of the cell membrane [202,203]. It was found that the NPC2 protein contains a functional cholesterol-binding site through which it exerts its high affinity for cholesterol and binds to the sperm membrane [204]. In humans, NPC2 is associated with cholesterol efflux from the sperm membrane during epididymal sperm maturation [205]. Its role in the removal of cholesterol from the lipid rafts in bull spermatozoa was suggested [206]. In mice, NPC2 is associated with the maintenance of cholesterol content in the sperm membrane [203]. These findings are consistent with results showing that higher content of NPC2 in poor-freezability semen leads to higher efflux of cholesterol [152]. This phenomenon affects the fluidity of the sperm plasma membrane [207] and increases the influx of calcium ions, which leads to impaired membrane integrity [207,208]. Based on NPC2’s cholesterol-binding ability, a possible protective mechanism of NPC2 in reducing boar sperm membrane lipid loss during cryopreservation was proposed [152]. NPC2 was specified as a potential freezability marker of semen [152]. These investigations show that cholesterol content in semen might be a potential marker of canine semen resistance to cold shock [25]. Moreover, the positive correlation between seminal plasma cholesterol and semen quality in humans suggests that the cholesterol profile of seminal plasma can be used as an indicator of semen quality [209].

Lipids exhibit significant complexity and dynamism, undergoing continuous alterations in response to physiological and environmental factors [210]. Lipidomics is a field of research on the complete lipid profile (lipidome) within a cell, tissue, or organism. It also provides a quantitative analysis of the identified lipids [211]. Lipidomics can be used to study signal processing, lipid metabolism, molecular mechanisms, and biomarker discovery [210]. Despite the growing popularity and development of lipidomic tools, a lack of knowledge remains. The lipid composition of canine semen has also not been fully explored. This study would shed new light on understanding the impact of various lipids on reproductive processes in dogs. Combined with other molecular biology methods, lipidomics can be used to highlight the lipid markers of male fertility [183]. The identified biomarkers can be subjected to further research and used to determine, in detail, the molecular basis of male fertility in the future.

### 3.2. Carbohydrates

The effect of carbohydrates on the reproductive functions of dogs has not been described in detail. The available literature is dominated primarily by reports on the addition of sugars (glucose, fructose) to semen extenders [212] or basal medium [213]. Sugars have long been incorporated into semen diluents as exogenous energy substrates, osmotic constituents, and cryoprotective agents [214]. The carbohydrate composition of dog semen (sperm and seminal fluids) and the impact of individual sugar components on semen quality need to be investigated to fill the existing lack of current knowledge. Research based on modern, accurate measurement methods may allow the identification of new sugar markers of dogs’ reproductive functions.

#### Heparin

Heparin belongs to the class of glycosaminoglycans [215]. Early studies showed that glycosaminoglycans are involved in events preceding the acrosome reaction [216,217] and stimulate acrosome reaction in sperm in vitro [218,219,220,221]. Interestingly, it has been speculated that sperm motility in the bitch reproductive tract is maintained by the glycosaminoglycans present in the uterine fluid, and the glycosaminoglycans in the oviductal and uterine fluids of estrous bitches are associated with in vivo capacitation of the dog sperm [222]. The role of heparin in reproductive processes can be considered in three ways: (a) heparin as an addition to the sperm capacitation medium; (b) heparin as a ligand bound to proteins present in the fluids of the reproductive system; and (c) heparin-binding proteins (HBPs) as a semen additive.

Heparin as an additive in the in vitro medium is effective in stimulating metabolic enzymes, prolonging sperm motility, and successfully inducing hyperactivation even in the sperm of asthenozoospermic dogs [223]. Additionally, Risopatrón et al., (2005) [224] showed the positive effect of the addition of heparin to the capacitating medium on the acrosome reaction of the canine sperm. The presence of heparin in an in vitro medium induces acceleration in the fertilizing ability of the bull epididymal sperm [225].

The ability of sperm to bind heparin is attributed to the presence of seminal plasma proteins. These proteins attach to the sperm surface (especially to lipids containing the phosphoryl-choline group) after ejaculation, which increases the number of heparin-binding sites on the cell [226,227,228]. The sperm is then allowed to respond to heparin [227]. Heparin binding to bovine seminal plasma proteins induces the efflux of cholesterol and phospholipids from sperm, leading to capacitation [229,230]. The binding of heparin to receptors localized in the proximal region of the sperm head results in alterations in the structure of the plasma membrane covering that specific area [216,231]. In consequence, this mechanism influences the modulation of the acrosomal reaction by zona pellucida glycoproteins [228]. Due to this, heparin is known as a potent enhancer of capacitation [218]. De Souza et al., (2006) [232] described HBPs of the canine seminal plasma. They showed that approximately 50% of the canine seminal plasma proteins are bound to heparin. Of the dog seminal plasma HBPs, CPSE is predominant [232], similar to the group of ZnBPs [6]. Moreover, there is a high homology between the CPSE and the PSA from the seminal plasma, and PSA has been identified as a heparin-binding protein. PSA is the protein marker for human prostate cancer [233,234,235]. Moreover, high concentrations of OPN were identified in the dog seminal plasma and recognized as HBP [77]. The functions of CPSE and OPN in canine reproduction are described above. The capacity of some proteins to bind several different ligands should be taken into account. It has been shown that boar seminal plasma ZnBPs have the ability to also bind heparin, which suggests that these proteins participate in events associated with the regulation of capacitation and acrosome reactions in boar sperm [7]. Seminal plasma HBPs play an essential role in the fertilization process, and any variations in the structures, content, and functions of these proteins may be associated with infertility [236]. Treatment of bull semen with seminal plasma HBPs improved sperm motility, viability, acrosome integrity, in vitro capacitation, and acrosome reaction by reducing the oxidative stress induced by cold shock and, consequently, damage to the sperm membrane [237].

This clearly indicates a positive effect of heparin and HBPs on dog semen quality and its participation in the fertilization process, suggesting that heparin and its binding proteins are distinguished as having marker potential.

### 3.3. Ions

The maintenance of proper sperm function is dependent on the balance of intracellular and extracellular ions and proper ion channel functioning. Sperm cells react rapidly to environmental fluctuations and the regulation of ion balance across their membrane has been shown to be of critical importance for sperm functional features such as motility and fertilization capacity [238]. Ion channels contribute significantly to modulating membrane potential through the maintenance of intracellular pH and osmotic balance [239]. Sperm motility acquisition, capacitation, acrosome reaction, hyperactivation, and transport in the female reproductive tract are ion channel-dependent processes, and the intracellular milieu is regulated by the activity of these ion channels [240]. The control of sperm membrane potential is essential for all these events, and it is regulated by complex signaling pathways that involve the activation of many ion transporters and ion channels present in the sperm membrane [241,242]. Thus, ion channel dysfunctions, interference with ion homeostasis, and sperm membrane potential alterations have important consequences for fertilization [238].

#### 3.3.1. Zinc Ions

Zinc is an essential biological component that plays significant roles in several processes, including signaling, enzymatic activities, and regulation of sexual maturation, ensuring the stability of membrane lipid bilayers and the quaternary structure of the chromatin, and mitochondrial oxidative stress [243,244,245,246]. Zinc ions regulate a number of events that are crucial for fertilization, such as normal testicular development, spermatogenesis, and sperm function [247]. Any alterations in zinc can cause abnormalities in the functioning of the organism and have been associated with several diseases in dogs (see review [248]).

It has been shown that most of the zinc in canine seminal plasma originates from the prostate [249]. Large amounts of free zinc in seminal plasma inhibit sperm motility, while reduced free zinc improves their motility [250,251]. Zinc released by the prostate into seminal plasma during ejaculation can be utilized in two different manners. It can be incorporated into sperm cells and thus perform a protective role towards sperm chromatin and membrane stabilization and sperm motility [252,253,254]. Free zinc ions can also bind to different protein ligands that are implicated in fertilization processes in the female reproductive tract [195,255,256,257], reducing the zinc fraction available for sperm cells [258]. Free zinc availability in semen is regulated by high molecular proteins, which bind this element and reduce its amount in seminal plasma [259]. The authors’ recent studies have shown that high molecular weight protein complexes of canine seminal plasma possess the ability to bind zinc ions, and this has a positive effect on the motility of spermatozoa stored at low temperatures [7].

Zinc–protein complexes were isolated from seminal plasma in canines and characterized for the first time by Mogielnicka-Brzozowska et al., (2012) [258]. This study showed that canine zinc-binding proteins (ZnBPs) occurred in their native state as high molecular aggregates. Mogielnicka-Brzozowska et al., (2015) [6] also showed that under denaturing and reducing conditions, these macromolecules disaggregate. Spectrometric analysis revealed that seminal plasma zinc ions play both structural and regulatory roles in the activity of CPSE, which is crucial for maintaining the normal function of prostate and sperm cells. Prostasomes have also been studied in the context of zinc. Prostasomes are small lipid membrane-confined extracellular vesicles in the mammalian reproductive tract fluids [260] with the ability to bind zinc ions [261,262,263]. Canine prostasome proteins have a very high affinity for zinc ions, which suggests the important role of the vesicles in the metabolism of zinc in the canine semen [263]. It was shown that the addition of prostasomes to dog ejaculates had a positive effect on sperm quality (sperm plasma membrane and acrosome integrity) during storage at low temperatures [263].

There are some works systematizing knowledge on the role of zinc in male reproduction [248,264,265,266,267]. However, zinc ions found in dog semen should be further investigated for their potential as markers of semen quality.

#### 3.3.2. Calcium Ions

Ca^2+^ levels are low in freshly ejaculated cells; however, during capacitation, Ca^2+^ levels increase due to the influx of extracellular calcium ions over the sperm plasma membrane after the opening of voltage-gated calcium channels [268]. Calcium ionophore is a substance that enables the transport of Ca^2+^ ions across the spermatozoa membrane by forming stable complexes with divalent cations [200]. Szász et al., (2000) [269] suggested that Ca^2+^-ionophore-induced membrane changes in dog sperm cells are valuable parameters for predicting the suitability of dog ejaculates for cryopreservation. Calcium ions regulate several reproductive processes and events that are crucial for proper fertilization: spermatogenesis, testosterone secretion, sperm capacitation, hyperactivation, motility, and acrosome reaction (in review [270]). Despite reports indicating the important role of calcium ions in the regulation of reproductive events in humans, knowledge of the function of calcium in the canine reproductive tract is very limited. With the current state of knowledge, it is difficult to define calcium as a BRF in dogs. However, according to studies conducted in humans, calcium ions should not be underestimated, and further research is needed to explain the molecular basis of their action as a potential BRF in dogs.

**Figure 4:**
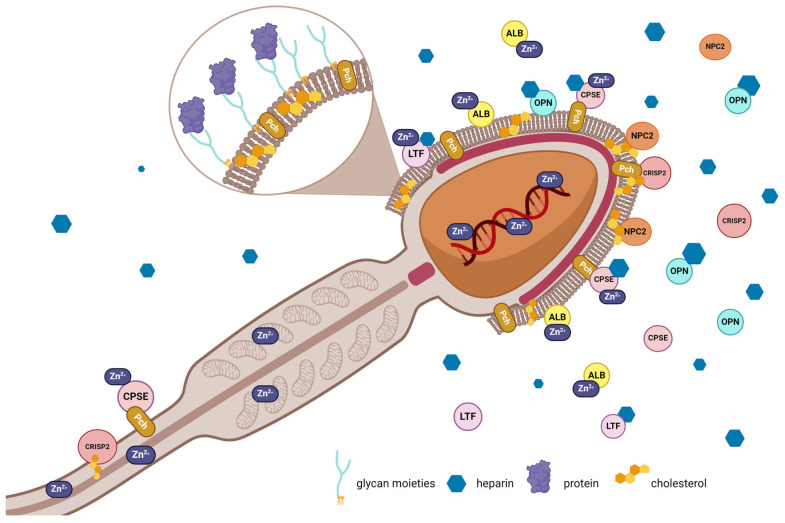
Lipids, carbohydrates, and ions and their binding proteins that are involved in canine sperm functions. ALB—albumin; CPSE—canine prostate-specific esterase; CRISP2—cysteine-rich secretory protein 2; LTF—lactotransferrin; NPC2—Niemann-Pick type C2 protein; OPN—osteopontin; Pch—phosphorylcholine; Zn^2+^—zinc ions. Created with BioRender.com.

Lipids, carbohydrates, and ions and their binding proteins that are involved in canine sperm functions are shown in Figure 4.

Table 2 contains a summary of the most promising lipid, carbohydrate, and ion molecular biomarkers of canine reproductive functions.

## 4. Differences in Biomarkers between Breeds

Purebred domestic dogs were created for backcrossing and inbreeding to determine desirable phenotypic or behavioral characteristics. These alterations may influence semen quality and impair biotechnologies of reproduction results [29]. Many canine breeds dealing with genetically specific diseases directly correlated with inbreeding in these breeds [271]. Some breeds may be more affected by reproductive impairments such as decreased semen quality or compromised fertility rates [272,273]. Araujo et al., (2022) [29] showed the influence of dog breed on the proteome of the spermatozoa and seminal plasma (for Golden Retriever, Great Dane, Bernese Mountain dog, and Maremmano-Abruzzese sheepdog). ALB was useful for the separation of the breeds based on spermatozoa proteins. Maremmano-Abruzzese Sheepdog had an increased abundance of ALB in the spermatozoa, and the Bernese Mountain dog had a low abundance of this protein. Tubulin alpha-3E chain, ACRBP, and tubulin alpha-3 chain were considered relevant in dogs’ seminal plasma. High abundances of the tubulin alpha-3E chain and the tubulin alpha-3 chain were found in the Maremmano-Abruzzese sheepdog and the Bernese Mountain dog, respectively. ACBP protein was found in higher abundance in the Bernese Mountain dog. According to the practical expertise of Araujo et al., (2022) [29], the Bernese Mountain dogs have low semen quality, which may be hypothesized as a pattern characteristic of this breed. The variations found between distinct canine breeds suggest that some cellular and molecular phenotypes associated with semen quality, intra-species cellular modulation, and motility could be breed-specific, similar to molecular markers of reproductive functions. Hallberg et al., (2024) [274] investigated the correlation between biomarkers for testicular cell function in the Bernese Mountain dog. Based on the markers discussed in our study, ALPL and CPSE levels vary between dogs based on semen quality and fertility. We found studies on the influence of breed and individual variation on the quality of frozen canine semen (Beagle, Schnauzer, Boxer, and Doberman) [275]. Based on the in vitro evaluation of the semen of different dog breeds, the quality of fresh semen does not show significant variation across the breeds studied; however, the integrity and viability of the sperm membrane and longevity of the thawed semen varies significantly both between individuals and between breeds. The canine spermatozoa cryotolerance varies based on the individual, and this variation may present some genetically inherited components that manifest as a characteristic of the seminal freezing of the breeds. These findings suggest that further studies on semen proteins that may be potential BRFs and functional investigations of breed-specific proteins in purebred dogs need to be performed to improve reproduction biotechnologies.

## 5. Characteristic Features of Molecular Biomarkers of Reproductive Processes

Most BRFs in dogs have certain unique characteristics. They are produced in the testicles, epididymis, or prostate, or are derived from blood and secreted into the reproductive organ fluids. These biomarkers interact with the sperm plasma membrane with the participation of proteins, lipids, carbohydrates, or ions. This interaction changes the functional characteristics of sperm. BRFs can be an integral part of sperm’s structure or may only be temporarily attached and detached from it, influencing sperm physiology. These biomarkers may also have functions associated with antioxidant properties. The levels of BRFs in semen or fluids surrounding sperm can vary depending on the physiological characteristics of the cells or reproductive organs. The content of molecular biomarkers directly or indirectly affects the condition of reproductive tissues or semen quality parameters, which in turn affects reproductive success. The levels of biomarkers can be measured accurately and reproducibly.

## 6. Conclusions

This review systematizes current knowledge that could serve as a starting point for developing practical tests for the use of biomarkers of canine reproductive functions and their predictive value for assisted reproductive technique outcomes and semen preservation. The literature review allowed for the systematization of the features of a typical molecular marker of reproductive processes in dogs. From the entire pool of molecular markers of reproductive functions in dogs, it is possible to distinguish a pool of markers that can be used with great probability in the future to improve reproductive processes in dogs, including semen conservation. Their functions include improving sperm capacitation, the acrosomal reaction, and sperm motility. They may be potential biomarkers of prostate and testicular cancer. There are markers whose expressions were correlated with the dog’s age. The addition of these biomarkers to the canine spermatozoa before or after preservation techniques preserves sperm viability and function, preventing cold shock consequences. Some described substances may be predictive biomarkers for the success of ART, such as IVF and ICSI. However, each type of marker must be treated individually in relation to these matters and this requires further in-depth research.

## Figures and Tables

**Table 1:** Summary of the most promising protein molecular biomarkers of canine reproductive functions described in the review. T—testis; EP — epididymis; EF—epididymal fluid; ES—epididymal spermatozoa; S—ejaculated spermatozoa; SP—seminal plasma; P—prostate; BS—blood serum, CD—cytoplasmic droplets.

Biomarker	Origin of Biomarker	Influence on Reproductive Functions	Implementation in Clinical/Breeding Practice	References
Well-studied proteins/highly abundant proteins				
AKAP4/ProAKAP4 (A-kinase anchoring protein)	T, S	- regulation of spermatogenesis - regulation of sperm capacitation - regulation of sperm motility	- biomarkers of capacitation status - associated with good-quality of spermatogenesis - involved in the regulation of spermatozoa motility - translation of semen quality markers into a practical tool (such as 4MID^®^ kits, 4BioDx, Lille, France)	[14,16,17,21]
ALBs (Albumins)	BS, T, EF, ES, SP, P	- preserves sperm viability - improves sperm capacitation and acrosome reaction - involved in zona pellucida penetration - regulates sperm motility - antioxidant properties	- The addition of heterogenous bovine serum albumin to the ejaculated canine spermatozoa preserves sperm viability and function - -improves sperm capacitation and acrosome reaction	[28,29]
ALPL (Alkaline phosphatase)	CD, BS, EP, SP	- dephosphorylation enzyme - transport organic molecules across biological membranes	- potential biomarker for prostate and testicular cancer - ALPL inhibitors can reduce the growth and invasion of cancer cells—measurement of ALPL enzymatic activity in the canine SP has been used in the diagnosis of incomplete ejaculation or azoospermia in dogs - a lower concentration of ALPL in the SP suggests bilateral obstruction of the vas deferens or epididymis	[37,39,40]
CLU (Clusterin)	T, ES, EF, SP, P	- positively influences sperm motility - inhibiting cell apoptosis - mediation of female tolerance to seminal antigens - sperm maturation processes	- an indicator of low semen quality because secretion of this protein is enhanced in case of cellular damage or heat shock	[42,46,47]
CPSE (Canine prostate-specific esterase)	S, SP, P	- multifunctional protein due to its zinc-binding and phosphorylcholine-binding properties - coat ejaculated spermatozoa, which could be implicated in sperm fertilization-related events	- marker of glandular secretion and serves as a promising diagnostic tool for non-neoplastic canine prostatic disorders	[4,6,40]
CRISP2 (Cysteine-rich secretory protein)	T, ES, S	- specifically regulates calcium flow through ryanodine receptors - is implicated in cell–cell adhesion and is capable of steroid binding	- decrease in CRISP2 content in sperm is associated with infertility in humans and horses - its content is low in the ES of very young and senile dogs	[26,53,54,59,60,61,62,63]
LTF (Lactotransferrin)	EP, SP, ES	- regulates the availability and catalytic activity of iron - has antibiotic properties in the reproductive system - able to bind lipopolysaccharides, heparin, glycosaminoglycans, and DNA, and ions like Mn^3+^, Co^3+^, Cu^2+^, and Zn^2+^	- its addition to a cryoprotective extender can significantly improve the function of frozen ram sperm	[64,65,66]
MMPs (Metalloproteinases)	E, SP, S	- contribute to the modification of the sperm membrane and the regulation of sperm maturation and storage - higher activation of proMMP-2, proMMP-9, and MMP-9 may be caused by an abnormal spermatogenesis process, whereas MMP-2 may benefit sperm motility and viability	- associations between MMP amounts in SP and parameters such as sperm concentration, motility, and morphology - the levels of MMPs and their tissue inhibitors are correlated with sperm motility and sperm DNA fragmentation in men - alterations in MMP expression or activity may indicate abnormalities in sperm function - MMPs have been proposed as predictive biomarkers for the success of assisted reproductive techniques (ART) such as in vitro fertilization (IVF) and intracytoplasmic sperm injection (ICSI)	[67,70,71,72,73]
OPN (Osteopontin)	T, EP	- implicated in various aspects of sperm function such as sperm maturation, motility, and capacitation, which are essential for successful fertilization - OPN localized to the post-acrosomal region on sperm membranes may participate in bovine fertilization by interacting with egg integrins	- lower OPN concentrations are linked to poorer sperm motility and morphology - it plays a major role in tumorigenesis, tumor invasion, and metastasis in prostate cancer	[75,76,78]
PTGDS (Prostaglandin-H2 D-isomerase)	EF, SP	- an enzyme that binds small non-substrate lipophilic molecules such as retinoids	- has a positive effect on human sperm’s progressive motility - PTGDS is present in greater abundance in the dog EF surrounding sperm showing good motility	[27,83,88]
Poorly studied/newly recognized/low-abundance proteins				
ACBP (Acrosin binding protein)	S	- improves sperm capacitation, acrosome reaction, and semen quality	- molecular marker for monitoring spermatogenesis in testicular tissues or germ cell development in vitro - marker for predicting boar sperm freezing capacity	[29,90,91]
ACTB (Actin binding protein)	ES, EF	- sperm capacitation and motility	- potential marker of canine ES aging - found in canine EF (highly abundant in EF of dogs with good sperm motility)	[26,27,92,93,94]
ELSPBP1 (Epididymal sperm-binding protein 1)	EP, ES	- binds to spermatozoa during their transit through the epididymis	- negatively correlated with bull fertility - is associated with the dead sperm population - is found in the epididymal spermatozoa, and its presence is age-dependent	[26,106,107]
LOC607874 (Cystatin domain-containing protein)	T, EP, ES, EF	- sperm motility	- potential biomarker of aging - its presence in EF has been associated with poor sperm motility	[26,27,118]
NPC2 (Niemann-Pick type C2 protein)	EP, S	- intracellular cholesterol transporter - gene is important in the etiology of Niemann-Pick type C disease - involved in cholesterol efflux from lysosomes	- higher content of NPC2 in poor-freezability semen leads to higher efflux of cholesterol - potential freezability marker of semen	[26,123,152]
OLFM4 (Olfactomedin 4)	S, EF, Epididymosomes	- regulates cellular growth, differentiation, and pathological processes	- the absence of its gene expression is associated with the progression of human prostate cancer - found in the EF of dogs, and is correlated with good sperm motility	[27,28,128]
TUBB (Tubulin)	SP, S	- associated with structural cell organization and flagella movement	- presence in SP may be correlated to sample handling	[29]
Antioxidant enzymes				
GPX5 (Glutathione peroxidase 5)	EP, SP, ES	- protects sperm from lipid peroxidation - works in the SOD/CAT system	- marker for resistance to cryodamage - the combination of GPX5 and SOD as an additive in the dilution extender of canine semen protects sperm viability and DNA integrity - addition of SOD, CAT, and GPX to the extender allows the preservation of semen quality in cold storage for both fertile and hypofertile dogs	[141,147,150,151]
SOD (Superoxide dismutase)	EP, SP, ES	- protects sperm from lipid peroxidation - works in the SOD/CAT system	as above	as above
CAT (Catalase)	SP	- enzymatic decomposition of hydrogen peroxide into water and oxygen - works in the SOD/CAT system	- addition of SOD and CAT to the dilution extender of canine semen improves sperm quality - addition of CAT to the extender allows the preservation of semen quality in cold storage for both fertile and hypofertile dogs	[119,149]

**Table 2:** Summary of the most promising non-protein molecular biomarkers of canine reproductive functions described in the review. EF—epididymal fluid; ES—epididymal spermatozoa; S—ejaculated spermatozoa; SP—seminal plasma; P—prostate.

Biomarker	Origin of Biomarker	Influence on Reproductive Functions	Implementation in Clinical/Breeding Practice	References
Lipids				
Saturated fatty acids (SFAs)				
Caprylic fatty acid	ES	- bactericidal properties - sperm membrane protection	- fatty acids supplementation for gamete manipulation techniques such as in vitro sperm maturation for immature canine spermatozoa - contraception, such as the use of inhibitors of the enzymes that are responsible for fatty acid biosynthesis, promoting lipid disruption of sperm - formulation of diluents and extenders, and improving cryopreservation protocols	[159,165,176]
Stearic fatty acid	ES, SP, S	- provides the energy for sperm motility and metabolism	as above	[159,167,168,169,170,171,176]
Palmitic fatty acid	SP	- positive effect on sperm production	as above	[159,172,176]
Monounsaturated fatty acids (MUFAs)				
Oleic fatty acid	SP	- regulation of sperm metabolism - positive effect on sperm motility and viability	- formulation of better diluents and extenders, and improving cryopreservation protocols	[159,174,176]
Polyunsaturated fatty acids (PUFAs)				
Docosahexaenoic acid (DHA)	ES, EF	- important for epididymal maturation - provides fluidity to the sperm plasma membrane affecting its integrity - positive effect on sperm cryotolerance	- fatty acids supplementation for gamete manipulation techniques such as in vitro sperm maturation for immature canine spermatozoa - contraception, such as the use of inhibitors of the enzymes that are responsible for fatty acid biosynthesis, promoting lipid disruption of sperm - formulation of better diluents and extenders, and improving cryopreservation protocols	[159,176,179,182]
Phospholipids				
Phosphatidylcholine	S	- sperm motility	- identification of asthenozoospermia	[164,170]
Phosphatidylethanolamine				
Plasmalogens				
Plasmenyl phosphatidylcholine (40:5)	S	- sperm motility - antioxidant properties	- formulation of diluents and extenders, and improving cryopreservation protocols	[8,164]
Plasmanyl phosphatidylcholine (40:4)				
Neutral lipids				
Cholesterol	SP	- maintaining the stability and fluidity of the cell membrane - protection against sperm damage induced by freeze/thaw processes	- potential marker of canine semen crytolerance - higher concentrations of cholesterol in the SP of ejaculates with good freezability - indicator of semen quality	[25,199,201,209]
Carbohydrates				
Heparin	oviductal and uterine fluids	- preceding and stimulation of sperm acrosome reaction in vitro - binding to SP proteins and performing different functions	- as an additive in the in vitro/capacitating medium: stimulates metabolic enzymes, prolongs sperm motility, and induces hyperactivation (even in the sperm of asthenozoospermic dogs), and has a positive effect on the acrosome reaction	[218,219,220,221,222,223,224,225]
Ions				
Zinc	SP, P	- sperm motility regulation - binding to SP proteins and performing different functions	- high levels of free zinc in SP inhibit sperm motility, while reduced free zinc levels improve sperm motility	[250,251,258]
